# Ethyl 3-amino-4-[(2-hy­droxy­ethyl)­amino]benzoate

**DOI:** 10.1107/S1600536810029077

**Published:** 2010-07-31

**Authors:** Natarajan Arumugam, Aisyah Saad Abdul Rahim, Shafida Abd. Hamid, Mohd Mustaqim Rosli, Hoong-Kun Fun

**Affiliations:** a School of Pharmaceutical Sciences, Universiti Sains Malaysia, 11800 USM, Penang, Malaysia; b Kuliyyah of Science, International Islamic University Malaysia, Jalan Sultan Ahmad Shah, Bandar Indera Mahkota, 25200 Kuantan, Pahang, Malaysia; c X-ray Crystallography Unit, School of Physics, Universiti Sains Malaysia, 11800 USM, Penang, Malaysia

## Abstract

In the crystal structure of the title compound, C_11_H_16_N_2_O_3_, mol­ecules are linked by one O—H⋯N and two N—H⋯O inter­molecular hydrogen bonds into a three-dimensional network, which incorporates *R*_2_^2^(14) and *R*_2_^2^(16) graph-set motifs.

## Related literature

For the biological activity of amino benzoic acid and benzimid­azole derivatives, see: Kumar *et al.* (2003 ▶); Stefan *et al.* (2002 ▶); Pan *et al.* (1999 ▶). For related structures, see: Narendra Babu *et al.* (2009 ▶); Abdul Rahim *et al.* (2010 ▶). For hydrogen-bond motifs, see: Bernstein *et al.* (1995 ▶).
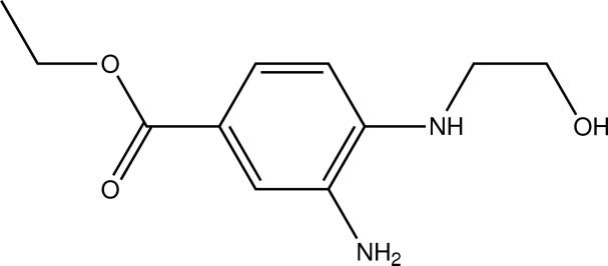


## Experimental

### 

#### Crystal data

C_11_H_16_N_2_O_3_
*M* *_r_* = 224.26
Monoclinic, 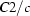

*a* = 23.2300 (5) Å
*b* = 14.5914 (4) Å
*c* = 7.5815 (1) Å
β = 108.931 (1)°
*V* = 2430.81 (9) Å^3^
*Z* = 8
Mo *K*α radiation
μ = 0.09 mm^−1^
*T* = 296 K
0.55 × 0.37 × 0.25 mm

#### Data collection

Bruker SMART APEXII CCD area-detector diffractometer
Absorption correction: multi-scan (*SADABS*; Bruker, 2009 ▶) *T*_min_ = 0.953, *T*_max_ = 0.978
26584 measured reflections
3739 independent reflections
2597 reflections with *I* > 2σ(*I*)
*R*_int_ = 0.035

#### Refinement

*R*[*F* ^2^ > 2σ(*F* ^2^)] = 0.045
*wR*(*F* ^2^) = 0.134
*S* = 1.02
3739 reflections
162 parameters
H atoms treated by a mixture of independent and constrained refinement
Δρ_max_ = 0.16 e Å^−3^
Δρ_min_ = −0.21 e Å^−3^

### 

Data collection: *APEX2* (Bruker, 2009 ▶); cell refinement: *SAINT* (Bruker, 2009 ▶); data reduction: *SAINT*; program(s) used to solve structure: *SHELXTL* (Sheldrick, 2008 ▶); program(s) used to refine structure: *SHELXTL*; molecular graphics: *SHELXTL*; software used to prepare material for publication: *SHELXTL* and *PLATON* (Spek, 2009 ▶).

## Supplementary Material

Crystal structure: contains datablocks global, I. DOI: 10.1107/S1600536810029077/lh5088sup1.cif

Structure factors: contains datablocks I. DOI: 10.1107/S1600536810029077/lh5088Isup2.hkl

Additional supplementary materials:  crystallographic information; 3D view; checkCIF report

## Figures and Tables

**Table 1:** Hydrogen-bond geometry (Å, °)

*D*—H⋯*A*	*D*—H	H⋯*A*	*D*⋯*A*	*D*—H⋯*A*
O1—H1*O*1⋯N2^i^	0.846 (15)	2.017 (15)	2.8628 (14)	178.4 (14)
N2—H1*N*2⋯O3^ii^	0.880 (19)	2.145 (19)	3.0083 (16)	167.0 (13)
N2—H2*N*2⋯O1^iii^	0.907 (15)	2.113 (15)	2.9800 (14)	159.7 (13)

Symmetry codes: (i) 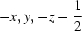; (ii) 
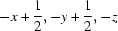
; (iii) 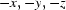.

**Fig. 1.**
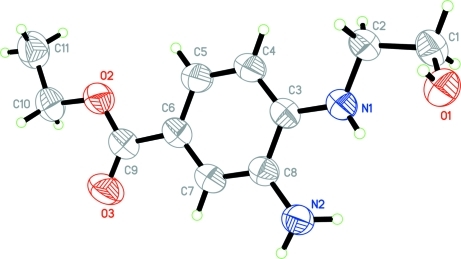
The molecular structure, showing 50% probability displacement ellipsoids and the atom-numbering scheme. Hydrogen atoms are shown as spheres of arbitrary radius.

**Fig. 2.**
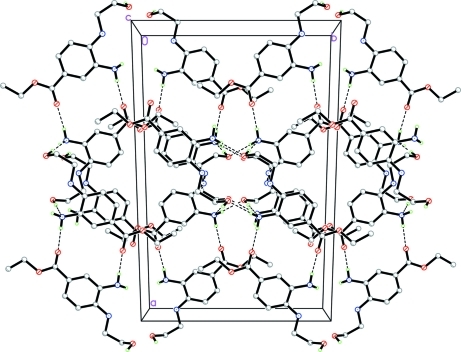
The crystal packing of (I) viewed along the c axis. Dashed lines indicate hydrogen bonds. H atoms not involved in the hydrogen bond interactions have been omitted for clarity.

## supplementary crystallographic information

### Comment

Amino benzoic acid derivatives are important intermediates for the synthesis of various heterocyclic compounds of pharmacological interests (Kumar *et al.*, 2003). The synthesis of novel benzimidazole derivatives such as 2-aminomethyl benzimidazoles (Stefan *et al.*, 2002) and oxobenzimidazoles (Pan *et al.*, 1999) are commonly accessed *via* aminobenzoic acid derivatives. As part of an ongoing study on such compounds, we present the crystal structure of the title compound (I), which was an intermediate in a synthesis.

In the title molecule (Fig. 1), the bond lengths and angles are within normal ranges and are similiar to those in related structures (Narendra Babu *et al.*, 2009; Abdul Rahim *et al.*, 2010). The benzoate group is essentially planar with a maximum deviation of -0.006 (1) for atom C4.

In the crystal structure,  molecules are linked by intermolecular N2—H1N2···O3^ii^, N2—H2N2···O1^iii^ and O1—H1O1···N2^i^ hydrogen bonds (see Table 1 for symmetry codes) into a three-dimensional network with R_2_^2^(14) and R_2_^2^(16) graph-set motifs (Bernstein *et al.*, 1995).

### Experimental

Ethyl 4-(2-hydroxyethylamino)-3-nitro-benzoate (0.5 g, 1.96 mmol), ammonium formate (0.4 g, 6.8 mmol) and palladium on carbon (250 mg) were mixed in ethanol. The reaction mixture was irradiated under microwave conditions at 373K for 2 minutes. After completion, the reaction mixture was filtered through celite and the filtrate was concentrated under reduced pressure to yield the crude product. The product was recrystallised from EtOAc to afford the title compound as colourless crystals.

### Refinement

N-bound and O-bound H atoms were located from a difference Fourier map and refined isotropically. The remaining H atoms were positioned geometrically [C-H = 0.93, 0.96 or 0.97 Å] and were refined using a riding model, with U_iso_(H) = xU_eq_(C), where x = 1.5 for methyl and 1.2 for all other atoms. A rotating model was used for the methyl group.

### Crystal data

**Table:**

C_11_H_16_N_2_O_3_	*F*(000) = 960
*M**_r_* = 224.26	*D*_x_ = 1.226 Mg m^−^^3^
Monoclinic, *C*2/*c*	Mo *K*α radiation, λ = 0.71073 Å
Hall symbol: -C 2yc	Cell parameters from 8941 reflections
*a* = 23.2300 (5) Å	θ = 2.8–30.6°
*b* = 14.5914 (4) Å	µ = 0.09 mm^−^^1^
*c* = 7.5815 (1) Å	*T* = 296 K
β = 108.931 (1)°	Block, colourless
*V* = 2430.81 (9) Å^3^	0.55 × 0.37 × 0.25 mm
*Z* = 8	

### Data collection

**Table:**

Bruker SMART APEXII CCD area-detector diffractometer	3739 independent reflections
Radiation source: fine-focus sealed tube	2597 reflections with *I* > 2σ(*I*)
graphite	*R*_int_ = 0.035
φ and ω scans	θ_max_ = 30.6°, θ_min_ = 1.7°
Absorption correction: multi-scan (*SADABS*; Bruker, 2009)	*h* = −32→33
*T*_min_ = 0.953, *T*_max_ = 0.978	*k* = −20→20
26584 measured reflections	*l* = −10→10

### Refinement

**Table:**

Refinement on *F*^2^	Primary atom site location: structure-invariant direct methods
Least-squares matrix: full	Secondary atom site location: difference Fourier map
*R*[*F*^2^ > 2σ(*F*^2^)] = 0.045	Hydrogen site location: inferred from neighbouring sites
*wR*(*F*^2^) = 0.134	H atoms treated by a mixture of independent and constrained refinement
*S* = 1.02	*w* = 1/[σ^2^(*F*_o_^2^) + (0.0626*P*)^2^ + 0.4393*P*] where *P* = (*F*_o_^2^ + 2*F*_c_^2^)/3
3739 reflections	(Δ/σ)_max_ < 0.001
162 parameters	Δρ_max_ = 0.16 e Å^−^^3^
0 restraints	Δρ_min_ = −0.21 e Å^−^^3^

### Special details

**Table:**

Geometry. All esds (except the esd in the dihedral angle between two l.s. planes) are estimated using the full covariance matrix. The cell esds are taken into account individually in the estimation of esds in distances, angles and torsion angles; correlations between esds in cell parameters are only used when they are defined by crystal symmetry. An approximate (isotropic) treatment of cell esds is used for estimating esds involving l.s. planes.
Refinement. Refinement of F^2^ against ALL reflections. The weighted R-factor wR and goodness of fit S are based on F^2^, conventional R-factors R are based on F, with F set to zero for negative F^2^. The threshold expression of F^2^ > 2sigma(F^2^) is used only for calculating R-factors(gt) etc. and is not relevant to the choice of reflections for refinement. R-factors based on F^2^ are statistically about twice as large as those based on F, and R- factors based on ALL data will be even larger.

### Fractional atomic coordinates and isotropic or equivalent isotropic displacement parameters (Å^2^)

**Table:**

	*x*	*y*	*z*	*U*_iso_*/*U*_eq_	
O1	−0.07544 (4)	0.04108 (6)	−0.13906 (13)	0.0595 (2)	
O2	0.17663 (4)	0.52426 (6)	0.03582 (14)	0.0667 (3)	
O3	0.24606 (4)	0.41940 (7)	0.03351 (18)	0.0826 (3)	
N1	0.01804 (4)	0.17082 (8)	0.01775 (14)	0.0544 (2)	
N2	0.13085 (5)	0.11676 (7)	0.00330 (14)	0.0540 (2)	
C1	−0.07977 (6)	0.10608 (9)	−0.00450 (17)	0.0581 (3)	
H1A	−0.0643	0.0789	0.1186	0.070*	
H1B	−0.1223	0.1212	−0.0277	0.070*	
C2	−0.04490 (5)	0.19272 (8)	−0.00811 (16)	0.0521 (3)	
H2A	−0.0627	0.2237	−0.1266	0.063*	
H2B	−0.0472	0.2336	0.0903	0.063*	
C3	0.05994 (5)	0.23731 (8)	0.01945 (15)	0.0490 (3)	
C4	0.04734 (5)	0.33047 (8)	0.02461 (19)	0.0600 (3)	
H4A	0.0091	0.3487	0.0258	0.072*	
C5	0.09038 (6)	0.39616 (9)	0.0280 (2)	0.0623 (3)	
H5A	0.0809	0.4579	0.0308	0.075*	
C6	0.14783 (5)	0.37052 (8)	0.02717 (16)	0.0539 (3)	
C7	0.16117 (5)	0.27727 (8)	0.02449 (15)	0.0518 (3)	
H7A	0.1997	0.2597	0.0254	0.062*	
C8	0.11884 (5)	0.21078 (8)	0.02050 (14)	0.0479 (2)	
C9	0.19533 (5)	0.43832 (9)	0.03180 (17)	0.0575 (3)	
C10	0.21965 (6)	0.59737 (9)	0.0476 (2)	0.0668 (3)	
H10A	0.2336	0.5963	−0.0598	0.080*	
H10B	0.2546	0.5910	0.1595	0.080*	
C11	0.18689 (8)	0.68429 (11)	0.0526 (3)	0.0838 (5)	
H11A	0.2149	0.7347	0.0733	0.126*	
H11B	0.1697	0.6817	0.1519	0.126*	
H11C	0.1550	0.6926	−0.0640	0.126*	
H1O1	−0.0924 (7)	0.0638 (11)	−0.246 (2)	0.075 (4)*	
H1N1	0.0238 (6)	0.1165 (11)	−0.0138 (19)	0.065 (4)*	
H1N2	0.1691 (8)	0.1076 (10)	0.012 (2)	0.072 (4)*	
H2N2	0.1176 (7)	0.0761 (10)	0.072 (2)	0.071 (4)*	

### Atomic displacement parameters (Å^2^)

**Table:**

	*U*^11^	*U*^22^	*U*^33^	*U*^12^	*U*^13^	*U*^23^
O1	0.0687 (5)	0.0560 (5)	0.0529 (5)	0.0008 (4)	0.0184 (4)	0.0009 (4)
O2	0.0536 (5)	0.0594 (5)	0.0905 (7)	−0.0071 (4)	0.0280 (4)	−0.0010 (4)
O3	0.0551 (5)	0.0787 (7)	0.1247 (9)	−0.0041 (4)	0.0441 (5)	−0.0072 (6)
N1	0.0481 (5)	0.0538 (6)	0.0645 (6)	−0.0011 (4)	0.0226 (4)	−0.0009 (4)
N2	0.0508 (5)	0.0569 (6)	0.0567 (6)	0.0036 (4)	0.0210 (4)	−0.0002 (4)
C1	0.0572 (6)	0.0655 (7)	0.0577 (7)	−0.0079 (5)	0.0270 (5)	−0.0043 (5)
C2	0.0481 (6)	0.0587 (6)	0.0534 (6)	−0.0015 (5)	0.0218 (5)	−0.0029 (5)
C3	0.0452 (5)	0.0574 (6)	0.0461 (5)	−0.0014 (4)	0.0172 (4)	−0.0015 (4)
C4	0.0462 (6)	0.0590 (7)	0.0794 (8)	0.0021 (5)	0.0266 (6)	−0.0004 (6)
C5	0.0533 (6)	0.0541 (7)	0.0839 (8)	0.0020 (5)	0.0285 (6)	−0.0005 (6)
C6	0.0475 (6)	0.0594 (7)	0.0575 (6)	−0.0028 (5)	0.0207 (5)	−0.0018 (5)
C7	0.0436 (5)	0.0638 (7)	0.0501 (6)	0.0018 (5)	0.0180 (4)	−0.0015 (5)
C8	0.0474 (5)	0.0561 (6)	0.0411 (5)	0.0021 (4)	0.0154 (4)	−0.0011 (4)
C9	0.0510 (6)	0.0643 (7)	0.0600 (7)	−0.0028 (5)	0.0219 (5)	−0.0024 (5)
C10	0.0603 (7)	0.0693 (8)	0.0737 (8)	−0.0151 (6)	0.0257 (6)	−0.0026 (6)
C11	0.0976 (11)	0.0696 (9)	0.0974 (12)	−0.0124 (8)	0.0499 (9)	−0.0071 (8)

### Geometric parameters (Å, °)

**Table:**

O1—C1	1.4203 (15)	C3—C4	1.3938 (17)
O1—H1O1	0.844 (17)	C3—C8	1.4194 (15)
O2—C9	1.3306 (16)	C4—C5	1.3792 (17)
O2—C10	1.4447 (15)	C4—H4A	0.9300
O3—C9	1.2065 (15)	C5—C6	1.3880 (16)
N1—C3	1.3714 (14)	C5—H5A	0.9300
N1—C2	1.4465 (14)	C6—C7	1.3972 (18)
N1—H1N1	0.851 (15)	C6—C9	1.4738 (17)
N2—C8	1.4144 (15)	C7—C8	1.3747 (16)
N2—H1N2	0.880 (17)	C7—H7A	0.9300
N2—H2N2	0.907 (16)	C10—C11	1.486 (2)
C1—C2	1.5064 (17)	C10—H10A	0.9700
C1—H1A	0.9700	C10—H10B	0.9700
C1—H1B	0.9700	C11—H11A	0.9600
C2—H2A	0.9700	C11—H11B	0.9600
C2—H2B	0.9700	C11—H11C	0.9600
			
C1—O1—H1O1	107.8 (11)	C4—C5—H5A	119.8
C9—O2—C10	118.22 (10)	C6—C5—H5A	119.8
C3—N1—C2	121.87 (10)	C5—C6—C7	118.72 (11)
C3—N1—H1N1	119.1 (10)	C5—C6—C9	122.18 (11)
C2—N1—H1N1	114.3 (10)	C7—C6—C9	119.10 (10)
C8—N2—H1N2	111.4 (10)	C8—C7—C6	121.82 (10)
C8—N2—H2N2	117.9 (9)	C8—C7—H7A	119.1
H1N2—N2—H2N2	112.2 (13)	C6—C7—H7A	119.1
O1—C1—C2	112.55 (9)	C7—C8—N2	121.69 (10)
O1—C1—H1A	109.1	C7—C8—C3	119.27 (10)
C2—C1—H1A	109.1	N2—C8—C3	118.86 (10)
O1—C1—H1B	109.1	O3—C9—O2	122.72 (12)
C2—C1—H1B	109.1	O3—C9—C6	124.60 (12)
H1A—C1—H1B	107.8	O2—C9—C6	112.68 (10)
N1—C2—C1	109.76 (10)	O2—C10—C11	106.38 (11)
N1—C2—H2A	109.7	O2—C10—H10A	110.5
C1—C2—H2A	109.7	C11—C10—H10A	110.5
N1—C2—H2B	109.7	O2—C10—H10B	110.5
C1—C2—H2B	109.7	C11—C10—H10B	110.5
H2A—C2—H2B	108.2	H10A—C10—H10B	108.6
N1—C3—C4	122.37 (10)	C10—C11—H11A	109.5
N1—C3—C8	119.14 (10)	C10—C11—H11B	109.5
C4—C3—C8	118.47 (10)	H11A—C11—H11B	109.5
C5—C4—C3	121.38 (11)	C10—C11—H11C	109.5
C5—C4—H4A	119.3	H11A—C11—H11C	109.5
C3—C4—H4A	119.3	H11B—C11—H11C	109.5
C4—C5—C6	120.33 (11)		
			
C3—N1—C2—C1	179.71 (10)	C6—C7—C8—C3	−0.13 (16)
O1—C1—C2—N1	−56.17 (13)	N1—C3—C8—C7	−179.22 (10)
C2—N1—C3—C4	9.84 (17)	C4—C3—C8—C7	−0.70 (16)
C2—N1—C3—C8	−171.70 (10)	N1—C3—C8—N2	5.47 (15)
N1—C3—C4—C5	179.39 (12)	C4—C3—C8—N2	−176.01 (10)
C8—C3—C4—C5	0.93 (18)	C10—O2—C9—O3	−1.65 (19)
C3—C4—C5—C6	−0.3 (2)	C10—O2—C9—C6	177.74 (10)
C4—C5—C6—C7	−0.54 (19)	C5—C6—C9—O3	179.09 (13)
C4—C5—C6—C9	−179.63 (12)	C7—C6—C9—O3	0.0 (2)
C5—C6—C7—C8	0.76 (17)	C5—C6—C9—O2	−0.29 (17)
C9—C6—C7—C8	179.87 (10)	C7—C6—C9—O2	−179.38 (10)
C6—C7—C8—N2	175.04 (10)	C9—O2—C10—C11	−179.15 (12)

### Hydrogen-bond geometry (Å, °)

**Table:**

*D*—H···*A*	*D*—H	H···*A*	*D*···*A*	*D*—H···*A*
O1—H1O1···N2^i^	0.846 (15)	2.017 (15)	2.8628 (14)	178.4 (14)
N2—H1N2···O3^ii^	0.880 (19)	2.145 (19)	3.0083 (16)	167.0 (13)
N2—H2N2···O1^iii^	0.907 (15)	2.113 (15)	2.9800 (14)	159.7 (13)

Symmetry codes: (i) −*x*, *y*, −*z*−1/2; (ii) −*x*+1/2, −*y*+1/2, −*z*; (iii) −*x*, −*y*, −*z*.
